# Impact of social network composition on cognitive decline: Digital Dementia Registry Bavaria (digiDEM Bayern)

**DOI:** 10.1371/journal.pone.0306447

**Published:** 2024-07-12

**Authors:** Lisa Laininger, Nikolas Dietzel, Elmar Graessel, Hans-Ulrich Prokosch, Peter L. Kolominsky-Rabas

**Affiliations:** 1 Interdisciplinary Center for Health Technology Assessment (HTA) and Public Health (IZPH), Friedrich-Alexander-Universität Erlangen-Nürnberg, Erlangen, Germany; 2 Center for Health Services Research in Medicine, Department of Psychiatry and Psychotherapy, University Hospital Erlangen, Friedrich-Alexander-Universität Erlangen-Nürnberg, Erlangen, Germany; 3 Chair of Medical Informatics, Department of Medical Informatics, Biometrics and Epidemiology, Friedrich-Alexander-Universität Erlangen-Nürnberg, Erlangen, Germany; Tokyo Metropolitan Institute of Geriatrics and Gerontology, JAPAN

## Abstract

**Background:**

Currently, there is no curative treatment for dementia. The implementation of preventive measures is of great importance. Therefore, it is necessary to identify and address individual and modifiable risk factors. Social isolation, defined through social networks, is a factor that may influence the onset and progression of the disease. The networks of older people are mostly composed of either family or friends. The aim of this study is to examine the influence of social isolation and network composition on cognition over the course of 12 months in people with cognitive impairment.

**Methods:**

Data basis is the multicentre, prospective, longitudinal register study ‘Digital Dementia Registery Bavaria—digiDEM Bayern‘. The degree of social isolation was assessed using the Lubben Social Network Scale- Revised (LSNS-R) and the degree of cognitive impairment using the Mini Mental State Examination (MMSE), conducted at baseline and after 12 months. Data were analysed using pre-post ANCOVA, adjusted for baseline MMSE, age, gender, education, living situation and Barthel-Index.

**Results:**

106 subjects (78.9 ± 8.2 years; 66% female) were included in the analysis. The mean MMSE score at baseline was 24.3 (SD = 3.6). Within the friendship subscore, risk for social isolation was highly prevalent (42.5%). Though, there was no difference between individuals with higher/ lower risk of social isolation within the friendship-network after adjusting for common risk factors in cognitive decline over time, F (1,98) = .046, p = .831, partial η^2^ = .000.

**Conclusion:**

The results of this study showed that the risk of social isolation from friends is very high among people with cognitive impairment. However, social isolation does not appear to have a bearing influence on the course of cognition. Nevertheless, it is important for people with cognitive impairment to promote and maintain close social contacts with friends.

## Introduction

In 2021, around 1.8 million people in Germany were living with dementia, prevalence increasing. Without a breakthrough in prevention or therapy, current estimates suggest that up to 2.8 million people aged 65 and older could be affected by 2050 in Germany alone [1]. Even though therapies for Alzheimer’s disease (AD) have recently been approved for specific subpopulations with AD [2], there is still a need for prevention and intervention strategies. Therefore, it is necessary to identify and address individual and modifiable risk factors [3]. Next to smoking, depression, lack of exercise, and diabetes, social isolation is a risk factor for dementia [4]. In general, social isolation is considered an overall risk factor for health, as people with weaker social ties showed an increased risk of poor self-rated health, musculoskeletal disorders, and depression [5] and have a higher risk of premature death [6]. In addition, according to calculations by Livingston et al. (2020), about 3.5% of dementia cases can be attributed to social isolation. This is almost as many as hypertension, diabetes, and obesity combined [4]. Furthermore, the results of two systematic reviews support these findings and highlight the link between social isolation or low social contact and cognitive function as well as cognitive decline [7, 8].

Social isolation is a critical public health problem that is highly prevalent in a German age cohort (32.3%) [9]. Social isolation, defined as infrequent social interactions or the complete absence of social ties [13], is usually conceptualised in terms of social networks. Social network refers to the relationships and connections in a person’s life [10]. A person’s network is categorized based on structural components (i.e. size and frequency) and functional components (i.e. closeness and quality) [11]. Amongst older people, the most relevant networks are composed of either family or friends. These networks usually differ in their functional and structural characteristic [12]. Notably, while the functional components contribute to perceived social support, the structural components of an individual’s network are often used to define the objective state of social isolation [13]. There are numerous reasons why the risk of social isolation increases with age. According to a recent publication of Takahashi et al. (2020), however, a variety of modifiable and non-modifiable risk factors can play an influential role, including gender, age, living with others and lack of participation in community groups [14].

Previous studies have shown that the extent of social isolation in people without cognitive impairment can significantly affect cognitive decline [9, 15, 16]. The well-conducted study by Röhr et al. (2020) showed that socially isolated people not only had lower cognitive function, but that a decrease in social networks was also associated with a decline in cognition over time [9].

In people with cognitive impairment or dementia, the relationship between social networks and cognitive trajectories is less clear. To our knowledge, there are only a few studies that focus on the relationship between social network and cognitive outcomes in people with cognitive impairment [17, 18]. Moreover, the emphasis is laying more on size of social network, frequency of contacts and closeness alone. There is also a lack of longitudinal data. However, these findings are needed to assess the relationship between social isolation and cognition. The aim of this study is to examine the influence of social network and network composition on cognition over the course of twelve months in people with cognitive impairment.

## Methods

### Study design

This study is part of the ongoing project ‘Digital Dementia Registry Bavaria–digiDEM Bayern’. digiDEM Bayern is a multicentre, prospective, longitudinal register study conducted in all administrative regions of Bavaria. The detailed methodology of the project is described elsewhere [19, 20].

### Study population

Participants are people with mild cognitive impairment (MCI) and people with mild or moderate dementia, who are in the following collectively referred to as people with cognitive impairment. To identify eligible participants, people have to undergo a screening based on the Mini-Mental State Examination (MMSE) and Montreal Cognitive Assessment (MoCA) prior to inclusion. Inclusion criteria for people with dementia are a MMSE value between 15–23 points, living in their home environment and having their primary residence in Bavaria. If the MMSE value is >23 points, the MoCA is conducted additionally to identify people with MCI [21].If so, the range for inclusion is 0–23 points [22]. All types of dementia and all age groups are included. People with an MMSE value between 0 and 14 points or a MoCA value >23 points, people who live in a nursing home, people with psychiatric diagnoses (e.g. depression, schizophrenia, addictions) or aphasia, and people who are deaf or blind are excluded. If available, a family caregiver is included with the person with cognitive impairment [19].

### Recruitment and data collection

Participants were recruited by specially trained research partners in all seven Bavarian administrative districts, starting on 1 July 2020. Recruitment will continue until 31 December 2024. Research partners are institutions that are specialized and have experience in the management and care of people with cognitive impairment and their family caregivers is conducted through standardized face-to-face interviews using a web-based data entry system [19, 20].

### Data protection and ethics

digiDEM Bayern collects and stores all personal data separately from the registry data on different stand-alone systems to ensure high protection standards of personal data and privacy. The project is performed following the ethical standards of the Declaration of Helsinki and obtained ethical approval from the Ethics Committee of the Medical Faculty of the FAU (application number: 253_20 B). Informed consent from the participants or their authorized representative is acquired electronically before start of screening and study inclusion. The declarations of consent are stored digitally in the REDCap registry software. Participation is voluntary [19, 20].

### Measurements

Sociodemographic data were collected at baseline. Six covariates were controlled for in the analysis such as age (years), gender, education and living situation, which are known covariates for cognition in later life [23–26]. Education was categorised into low, medium and high education according to the International Standard Classification of Education [27]. The living situation was obtained by asking whether the person does live alone or with someone, e.g. partner, children, siblings or others.

In addition, overall score of the Barthel Index was included in the analysis. The Barthel Index (BI) is a widely used tool to measure the ability to perform activities of daily living (ADL). A high score indicates a higher degree of independence in carrying out basic ADL´s such as eating, getting dressed, personal hygiene and using the toilet [28, 29]. A low score may suggest a lack in physical ability and mobility, which can also affect the degree of social isolation [30]. The maximum score is 100.

Social isolation was measured using the Lubben Social Network Scale-Revised (LSNS-R), a relatively brief, standardized quantitative measure to assess social isolation in older adults by measuring the number and frequency of contacts with friends and family as well as social support received by them [31]. The questionnaire consists of twelve questions in total, six of which cover kin-social ties and six comparable questions assessing non-kinship ties [31]. Each of the twelve LSNS-R questions is scored from 0 to 5 like a Likert-scale, and the total score ranges from 0 to 60. Higher scores indicate a lower risk for social isolation [32]. Individuals with a score below 15 are considered to be at a higher risk for social isolation. This cut-off value is based on independent extrapolations from previous publications that used a cut-off value for the shorter version of the LSNS-R, the Lubben Social Network Scale-6. The total score of the LSNS-R was divided into quartiles. For LSNS-R scores that fall within the lowest quartile, the individuals were categorised as having a poor social network. The individuals in the remaining three quartiles were to have an "average/good" social network. The same procedure was also used for the partial values for friends and relatives [18].

After the initial screening for cognitive impairment, using MMSE and MoCA, only the MMSE was used to record global cognition over time. The MMSE scores provided a measure for change in cognitive function [33]. Higher scores indicate better overall cognitive function. The maximum score is 30.

### Statistical analysis

#### Descriptive statistics

Descriptive data is reported for the overall sample at baseline and separately for those who are at a higher risk for social isolation (LSNS-R score below 15) and those with a low risk for social isolation. Continuous variables are presented as mean (M), standard deviation (SD), and standard error (SE) for adjusted variables, whilst categorical variables are presented as frequencies and percentages. Between group differences were compared using t-tests and chi-square tests. To assess the effect of social isolation on cognition after twelve months without adjusting for covariates, a one-way analysis of variance (ANOVA) was conducted.

#### ANCOVA

A pre-post ANCOVA (Analysis of Covariance) was used to examine the effect of social isolation (independent variable) on cognition (dependent variable) across two-time points while controlling for the potential influence of five covariates as well as cognition at baseline. The ANCOVA model was chosen to assess the main effects and interactions between the independent variable and measurement time points while adjusting for the covariates age (years), gender, living situation (solitary or not solitary) and level of education that are known to have a significant influence on cognition [23–25]. Since basic cognitive skills may impact the relationship between social integration and dementia risk, baseline MMSE scores were also included as a covariate [3]. Furthermore, the model was adjusted for overall BI score at baseline. Data analysis was conducted using IBM Statistical Package for the Social Sciences (SPSS) version 28.

## Results

In the period between August 2020 and January 2024 in total 106 participants were included in the study. The mean age of the participants was 78.9 (SD = 8.2) years and n = 70 (66.0%) were female. The mean MMSE score measured at baseline was 24.3 (SD = 3.6), and the mean LSNS-R score at baseline was 24.1 (SD = 10.3). More than half of the participants reported that they did not live alone (61.3%). The overall BI score at baseline was 86.5 (SD = 19.5). The mean social network score was 9.4 (SD = 5.0) for socially isolated and 27.7 (SD = 7.7) for socially integrated participants (t = 10.350, p < .001). 42.5% of participants showed an increased risk of social isolation from friends, while the risk of social isolation from family was only 17%. The baseline characteristics of the analytic sample are shown in **Table 1.**

**Table 1:** Comparison of demographic data among whole study population and the groups with a poor and an average social network.

Variable	Total (n = 106)	Poor social network (n = 21)	Average/good social network (n = 85)	t(df) or X^2^(df), p-Value
**Age (years), M (SD)**	78.9 (8.2)	77.4 (8.3)	79.3 (8.2)	t(104) = -.955, p = .342
**Gender, n (%)**				X^2^(1) = .924, p = .336
male	36 (34%)	9 (42.9%)	27 (31.8%)	
female	70 (66%)	12 (57.1%)	58 (68.2%)	
**Education, n (%)**				X^2^(2) = .609, p = .737
low	15 (14.2%)	2 (9.5%)	13 (15.3%)	
medium	70 (66.0%)	14 (66.7%)	56 (65.9%)	
high	21 (19.8%)	5 (23.8%)	16 (18.8%)	
**Living situation, n (%)**				X^2^(1) = 1.128, p = .288
solitary	41 (38.7%)	6 (28.6%)	35 (41.2%)	
not solitary	65 (61.3%)	15 (71.4%)	50 (58.8%)	
**Baseline BI score, M (SD)**	86.5 (19.5)	78.8 (21.3)	88.4 (18.7)	t(104) = -2.048, p = .034*
**Cognition basline, M (SD)**	24.3 (3.6)	22.8 (4.3)	24.7 (3.3)	t(104) = -2.216, p = .029*
**LSNS-R, M (SD)**	24.1 (10.3)	9.4 (5.0)	27.7 (7.7)	t(104) = -10.350, p < .001*
**Subscore Friends, M (SD)**	9.0 (6.6)	3.7 (3.5)	10.3 (6.5)	t(104) = -4.494, p < .001*
Poor social network, n (%)	45 (42.5%)			
Good/average social network, n (%)	61 (57.5%)			
**Subscore Family, M (SD)**	15.1 (7.0)	5.7 (5.3)	17.4 (5.3)	t(104) = -9.093 p < .001*
Poor social network, n (%)	18 (17%)			
Good/average social network, n (%)	88 (83%)			

Note: BI: Barthel Index; LSNS-R: Lubben Social Network Scale- Revised

*p-Value < 0,05

### Group-differences between good and poor social network and cognition

A one-way ANOVA was conducted to investigate whether there was a difference in cognition after twelve months (measured with MMSE) depending on the degree of social isolation at baseline. There was no statistically significant difference in cognition between the poor and good/average social network groups, F (1, 104) = 1.180, p = .280, partial η^2^ = .011. After adjusting for baseline MMSE scores, age, gender, education level, housing situation and BI score, no statistically significant difference in cognition at twelve months was found between the two groups either F(1, 98) = 1.492, p = .225, partial η^2^ = .015.

However, independent of the risk of social isolation, there was a separate, significant effect of MMSE, F (1, 98) = 108.536, p < .001, partial η^2^ = .526, and BI score at baseline, F (1, 98) = 5.351, p = .023, partial η^2^ = .052, when the other predictors were held constant (See **Table 2**).

**Table 2:** Adjusted group-differences between good and poor social network and cognition.

LSNS-R Score								
unadjusted Model					adjusted Model			
Variable	df	F	p-Value	η^2^	df	F	p-Value	η^2^
**Group**	1	1.180	.280	.011	1	1.492	.225	.015
**MMSE baseline**	-	-	-	-	1	108.536	< .001*	.526
**Age**	-	-	-	-	1	1.275	.262	.013
**Sex**	-	-	-	-	1	.136	.713	.001
**Education**	-	-	-	-	1	.115	.735	.001
**Living situation**	-	-	-	-	1	2.660	.106	.026
**Baseline BI**	-	-	-	-	1	5.351	.023*	.052
**Error**	104				98			

Note:dependent Variable: cognition (MMSE score) after 12 months, MMSE: Mini-Mental State Examination, BI: Barthel Index, LSNS-R: Lubben Social Network Scale- Revised

*p-Value < 0,05

### Group differences between good and poor social networks within friends and family and impact on cognition

The results of the ANOVA showed a significant difference in cognition between the groups with a higher and lower risk of social isolation based on the LSNS-R subscore friends, F (1, 104) = 8.477, p = .004, partial η^2^ = .075. However, when controlling for the covariates MMSE at baseline, age, gender, education, living situation and BI score, no significant difference between the groups was found F (1,98) =. 046, p = .831, partial η^2^ = .000. In addition, no statistically significant difference in cognition was found between the lower and higher risk group for social isolation based on the family subscore after controlling for the same covariates F (1, 98) = .355, p = .553, partial η^2^ = .004.

Furthermore, the baseline score on the MMSE and the score on the BI were found to be significant predictors of the dependent variable in both sub-groups (See **Table 3**).

**Table 3:** Adjusted group-differences between a good and a poor social network and cognition divided by LSNS-R subscale scores for friends and family.

Subscore friends									Subscore family							
	unadjusted Model				adjusted Model				unadjusted Model				adjusted Model			
**Variable**	**df**	**F**	**p-Value**	**η^2^**	**df**	**F**	**p-Value**	**η^2^**	**df**	**F**	**p-Value**	**η^2^**	**df**	**F**	**p-Value**	**η^2^**
**Group**	1	8.477	.004*	.075	1	.046	.831	.000	1	.233	.631	.002	1	.355	.553	.004
**MMSE baseline**	-	-	-	-	1	99.729	< .001*	.504	-	-	-	-	1	106.396	< .001*	.521
**Age**	-	-	-	-	1	1.930	.168	.019	-	-	-	-	1	1.498	.224	.015
**Sex**	-	-	-	-	1	.090	.765	.001	-	-	-	-	1	.056	.813	.001
**Education**	-	-	-	-	1	.168	.683	.002	-	-	-	-	1	.143	.706	.001
**Living situation**	-	-	-	-	1	2.347	.129	.023	-	-	-	-	1	2.605	.110	.026
**Baseline BI**	-	-	-	-	1	4.581	.035*	.045	-	-	-	-	1	4.685	.033*	.046
**Error**	104				98				104				98			

Note: dependent Variable: cognition (MMSE score) after 12 months, MMSE: Mini-Mental State Examination, BI: Barthel Index, LSNS-R: Lubben Social Network Scale- Revised

*p-Value < 0,05

**Table 4** provides a comparison of the adjusted and unadjusted mean values across the three groups, divided into good and poor social networks. The results suggest that, given similar baseline characteristics, participants at higher risk of social isolation would also have higher MMSE scores at twelve months.

**Table 4:** Adjusted means for cognition in the different LSNS-R-Groups after 12 months.

Variable	LSNS-R Score			Subscore friends			Subscore Family		
	N	Unadjusted M (SD)	Adjusted M (SE)	N	Unadjusted M (SD)	Adjusted M (SE)	N	Unadjusted M (SE)	Adjusted M (SE)
**MMSE t12** **(poor social network)**	21	23.523 (4.203)	24.956 (.548)	45	23.111 (4.058)	24.414 (.382)	18	23.944 (4.399)	24.674 (.592)
**MMSE t12** **(good social network)**	85	24.554 (3.809)	24.199 (.262)	61	25.262 (3.526)	24.301 (.323)	88	24.432 (3.802)	24.283 (.259)

Note: MMSE: Mini-Mental State Examination, LSNS-R: Lubben Social Network Scale- Revised

## Discussion

To our knowledge, this is the first cohort study based on registry data to investigate the relationship between social isolation, network composition and cognition in people with cognitive impairment. The rate of people with a poor social network of friends was very high at 42.5%. A significant difference was found between participants with a poor and a good friendship network. This suggests that friendships may play an important role in cognitive decline.

In accordance with our study, Moormann et al. (2023) found a significant difference in the prevalence of social isolation between friends (44.3%) and family (21.8%) in the oldest-old [12]. Moreover, the risk of social isolation within the friend network was very high in our cohort (42.5%) and a difference between individuals with a good and a poor non-kinship network was observed. Recent studies have shown that friendship networks play an important role in cognitive trajectories. Balouch et al. (2019) demonstrated that a more extensive social network with close friends was associated with better global cognition in people with AD. Additionally, no evidence was found for the impact of family networks [17]. Furthermore, other studies also suggested that not only the size, quality, and frequency of contacts but also the specific composition (i.e. network of friends or families) could influence cognition and risk of dementia [34, 35].

This reflects the particular role of background and social network influence. Family and friends are the two most essential components of social networks among older adults [36]. The former are expected to be more voluntary, and the latter involuntary formed [11, 37]. These components also often differ in terms of their structural characteristics (e.g. network size and frequency of contact) and their functional characteristics (e.g. proximity and quality of relationships). The quality of relationships may also vary. Friendships, in general, have been shown to contribute more to well-being than family ties [11, 38]. These relationships enable them to engage in social activities and exchange information [39]. In addition, non-kinship networks can promote positive health behaviours (e.g. physical activity) [40] through normative guidance and lived example.

Another theory that may explain the protective function of friendship networks is that of cognitive reserve. The theory proposes that despite existing age-related brain pathology, individuals are able to develop different levels of resilience throughout their lifespan to maintain cognitive function or compensate for existing deficits [41–43]. Cognitive reserve can be developed through participation in social and cognitively stimulating activities. According to cognitive reserve theory, engaging in social networks and interacting with other people would provide mental stimulation and therefor slow down cognitive decline [15].

Although baseline BI and cognitive abilities were independent predictors, the level of risk of social isolation did not show an effect on cognition at twelve months. This contradicts the findings of a number of previous studies that have shown an association between social isolation and cognition [9, 15, 16, 44, 45]. This could be due to the fact that the interaction between cognition and social isolation is likely bi-directional [18]. Evidence shows that people with cognitive and physical health impairments have difficulties to maintain social relationships [46]. Therefore, poor social relationships may be a consequence of cognitive decline rather than a cause. Moormann at al. (2023) were able to demonstrate that cognitive function served as a protective factor against social isolation in relation to friends as well as family [12]. In addition, Dyer et al (2021) also found a significant association between onset severity of dementia and subsequent social network decline. These findings suggest that the relationship between social networks and dementia progress may act as a risk factor, with the risk of social isolation increasing as cognition decreases [18].

The novelty of this study is that it focuses on people with mild to moderate dementia and mild cognitive impairment (MCI), a population that has not been extensively studied in relation to social isolation and cognition. Even in the early stages of dementia or MCI, individuals often have difficulties interacting socially due to their symptoms. Cognitive deficits such as memory loss or problems remembering names, as well as changes in emotions and experiences, can affect the ability to behave and interact socially with others [47,48]. This reinforces the downward spiral that leads to decreased social participation while increasing social isolation, which ultimately exacerbates the risk of cognitive decline [48].

The findings of this study further support these theories, as the participants are already highly affected by a loss of their friendship network. With an average MMSE score of 24.3, the study population consists mainly of people with MCI and provides a valuable insight into the relationship between social isolation and cognitive decline. Nevertheless, it is not clear to what extent cognitive status affects the degree of social isolation. This should be further investigated in people with MCI to better understand the mechanisms underlying this interaction and the bi-directional nature of the relationship.

### Limitations

This study has some limitations. First, a potential selection bias cannot be completely eliminated as the decision-making process on which the multiple research partners include the participants may vary and cannot be presented in its entirety. Although the research partners are facilities that are specialised in the care and support of people with cognitive impairment and distributed over all seven Bavarian administrative regions, this study may not be representative of the overall population. Second, there is the possibility of an interview bias. Even though the survey instruments used are validated questionnaires and the research partners are trained and frequently retrained, it cannot be ruled out that because different interviewers are involved, the individual approach of these interviewers may be perceived differently by the respondents. Particularly in the case of the Lubben Social Network Scale, targeting the subjective experience of interpersonal relationships, a potentially sensitive topic, a more empathetic approach on the part of the respondent can possibly have an influence on the information provided. Thirdly, although longitudinal data formed the basis for the analysis, no causal relationship between social isolation and cognitive decline was statistically analysed. Lastly, although the LSNS-R captures the size, closeness, and frequency of social networks [31] there are some other factors that were unable to address such as social engagement and social activities, depression, and quality of networks may also influence state of cognition [9, 49–52]. Because individuals with depression were excluded from participation in the registry study, the risk of confounding is reduced.

## Conclusion

The results of this study showed that the risk of social isolation from friends is highly prevalent among people with cognitive impairment and the network of friends seem to play a role in cognitive decline over the course of twelve months. However, no difference was found when known risk factors were controlled for. Overall, the role of the extent of social isolation showed no influence on cognitive trajectories. Nevertheless, the role of friendship in old age and for people with cognitive impairment deserves special attention. The risk of not being able to maintain close, trusting relationships with people outside their family environment seems to be particularly high for this group of people. It appears that these relationships may be important in the maintenance of health-promoting factors and the prevention of age-related cognitive decline. In order to support people already affected by cognitive impairment to maintain existing friendship ties or even to establish new, non-kinship relationships, it is necessary to create target group-specific offers such as art or movement activity groups for people with and without dementia or cognitive impairment. Even though the findings of this study provide a great deal of knowledge on social network composition and its effect on cognitive decline in people with cognitive impairment, there is a demand for more in-depth research on this topic. Future studies should look more into the relationship between the quality of social networks and cognition to gain a more precise insight into the connection and influential role of network composition.
